# Identification of ionotrophic purinergic receptors in Huh-7 cells and their response towards structural proteins of HCV genotype 3a

**DOI:** 10.1186/1743-422X-8-431

**Published:** 2011-09-08

**Authors:** Sobia Manzoor, Muhammad Idrees, Javed Ashraf, Azra Mehmood, Sadia Butt, Kaneez Fatima, Haji Akbar, Irshad U Rehaman, Ishtiaq Qadri

**Affiliations:** 1 National Centre of Excellence in Molecular Biology, University of the Punjab, Lahore, Pakistan; 2 NUST Center of Virology and Immunology (NCVI), National University of Sciences and Technology (NUST), Islamabad 44000, Pakistan; 3 Margalla Institute of Health Sciences, Quaid-e-Azam Avenue Gulrez Phase-III, Rawalpindi, Pakistan

**Keywords:** Hepatitis C virus, Hepatocellular carcinoma, receptor, isoform

## Abstract

Hepatitis C virus (HCV) is a major health problem in developing countries including Pakistan. Chronic HCV infection results in progressive liver disease including fibrosis, cirrhosis, insulin resistance and eventually hepatocellular carcinoma (HCC). Ionotrophic purinergic (P2X) receptors are identified to involve in a spectrum of physiological and pathophysiological processes. However, the role of P2X receptors in HCV liver associated diseases still remains to be investigated. The current study was designed to identify the presence of P2X receptors in human liver cells. Furthermore, it investigates the response of P2X receptors towards HCV structural proteins (E1E2). To determine that how many isoforms of P2X receptors are expressed in human liver cells, human hepatoma cell line (Huh-7) was used. Transcripts (mRNA) of five different isoforms of P2X receptors were identified in Huh-7 cells. To examine the gene expression of identified isoforms of P2X receptors in presence of HCV structural proteins E1E2, Huh-7/E1E2 cell line (stably expressing HCV structural proteins E1E2) was used. The results showed significant increase (6.2 fold) in gene expression of P2X4 receptors in Huh-7/E1E2 cells as compared to control Huh-7 cells. The findings of present study confirmed the presence of transcripts of five different isoforms of P2X receptors in human liver cells and suggest that P2X4 receptors could be represented an important component of the purinergic signaling complex in HCV induced liver pathogenesis.

## Background

Hepatitis C virus (HCV) is blood borne pathogen which is a major cause of different liver associated diseases varying from an asymptomatic condition to hepatocellular carcinoma (HCC) [1-5]. It is estimated that 3.3% of the population globally and 10% of the Pakistani population is chronically infected with HCV [6,7]). The genome of HCV is 9.6-kb-long, which predetermines a polyprotein of about 3,010 amino acids and encodes 3 structural (core, E1, E2, p7) and 7 nonstructural (P7, NS2, NS3, NS4A, NS4B, NS5A, NS5B) proteins [3,8-10]. These viral proteins not only function in viral replication but also dysregulate a variety of cellular functions [1,11,12].

During their synthesis, HCV envelope proteins E1E2 are inserted into the membranes of endoplasmic reticulum (ER). Immunolocalization studies have revealed the presence of E1E2 in the ER [13]. Liver mitochondria from transgenic mice expressing the HCV proteins core, E1 and E2, showed a significant decrease in mitochondrial glutathione and reduced activity and increased reactive oxidative species (ROS) production from mitochondrial electron transport complex I. These results suggest that changes in mitochondrial glutathione and complex I inhibition induced by core, E1 and E2 is an important cause of the oxidative stress seen in chronic hepatitis C [14]. Therefore, HCV envelope glycoproteins (E1E2) are a good candidate to study the HCV induced ROS, very well known reported to play a key role in the development of hepatic and extrahepatic complications in HCV chronic infection [15]. The major HCV genotype in Pakistan is 3a, with a strong association between chronic HCV infection and HCC in Pakistani population correlated with genotype 3a [6,16,17].

Significant attention is presently being drawn towards the HCV modulated molecular pathways that lead to liver pathogenesis [18,19]. Although many of the signals that regulate induction of liver pathogenesis have been defined, however, the second messengers by whom these signals are transduced are not as well established [20]. Adenosine 5'-triphosphate (ATP) functions as potent autocrine/paracrine signaling molecule in hepatocytes [21]. ATP can be released to the extracellular milieu under pathophysiological conditions such as stress, hypoxia, mechanical and osmotic strain and increase in cell volume [21,22]. ATP mediates its various effects by binding to and activation of Purinergic receptors [23]. Purinergic receptors are classified into two main types, P1 and P2 [24]. P2 receptors are further classified into two major types: P2X (ionotropic), and P2Y (metabotropic). Different isoforms of P2X receptors, expressed on various cells are identified to involve in a spectrum of physiological and pathophysiological processes, including synaptic transmission, pain and touch perception, vasomotor responses, platelet aggregation, endothelial release of vasorelaxants, cell volume regulation, cell proliferation and mitogenesis, apoptosis, wound healing, restenosis, atherosclerosis, ischaemia, inflammation, collagen deposition, and fibrosis [25-27]. The present study was undertaken to identify the presence of various isoforms of P2X receptors in human hepatoma cell (Huh-7) and to evaluate the response of identified isoforms to HCV structural proteins E1E2. The findings of the current study demonstrated that each of these isoforms of P2X receptors respond differently to HCV structural proteins E1E2. This is one of the first studies which may open a new insight into the pathogenic mechanisms leading to HCV induced liver pathogenesis via Purinergic (P2X) receptor signaling.

## Methods

### Cell lines and Culture Conditions

The cell lines used in this study, Huh-7 and Huh-7/E1E2 were kindly provided by Dr. Zafar Nawaz (University of Miami, USA) and Dr. Muhammad Idrees (Incharge Molecular Virology Division, CEMB, university of the Punjab, Lahore, Pakistan respectively. The Huh-7/E1E2 cell line was derived from the parental Huh-7 cell line. The Huh-7/E1E2 cell line was a stable clone of Huh-7 cells transfected with and stably over-expressing HCV Proteins E1E2 (derived from local HCV isolates of genotype 3a) linked to the antibiotic selection marker G418 which was under the control of human cytomegalovirus promoter [28] Huh-7 cell line was cultured in Dulbecco Modified Eagle's Medium (DMEM) supplemented with 100 U/ml of penicillin and 100 ug/ml of streptomycin and 10% heat inactivated fetal bovine serum (complete DMEM medium). Huh-7/E1E2 cell line was maintained in complete medium containing 500 μg/ml G418. The medium was replaced every third day, and cells were passaged every 4-5 days. All cells were maintained at 37°C in a humidified environment containing 5% CO2 in a cell culture incubator.

### RNA Isolation, quantification, and cDNA Synthesis

Total RNA from Huh-7 and Huh-7/E1E2 cells was isolated using Gentra RNA Isolation Kit (Puregene, Minneapolis, MN 55441, USA), following the manufacturer's instructions. Each sample of isolated RNA was further treated with DNase (Fermentas) to remove any residual DNA present that could generate false -positive results. Quantity and quality of extracted RNA was assessed using NanoDrop^® ^(Spectrophotometer) (ND-1000). Quality of extracted RNA was also checked on agarose gel electrophoresis using 1% agarose gel, stained with ethidium bromide and photographed. To investigate the presence of isoforms of P2X receptors cDNA was synthesized with 1 μg and 5 μg of total isolated RNA using RevertAid™ H minus First Strand cDNA synthesis kit (Fermentas, Cat no.K1632). All the tubes, pipette tips and containers used in the RNA isolation and cDNA synthesis were either manufacturer certified RNAase free or were made RNAase free by treating with 1% DEPC treated water and were autoclaved before use.

### Detection of P2X receptor transcripts Using RT-PCR

Independent sense and antisense primers were designed for P2X6 from known cloned receptor sequences, available in GenBank using primer 3 software, and were synthesized by the core synthesis facility of the centre (CEMB). Previously published primer sequences were used for the remaining receptors, P2X1, P2X2, P2X3, P2X4, P2X5, and P2X6. Primer sequences are shown in Table 1. PCR conditions for amplification of P2X2, P2X3 and P2X5 were 90°C (10 min), 35 cycles [95°C (45 s), 60°C (45 s) and 72°C (45 s)], and 72°C (10 min), PCR conditions for P2X4 were 94°C for 45 s 35 cycles [94°C (1 min), 60°C (45 s) and 72°C (1 min)], and 72°C 10 min. PCR amplifications were repeated using 10 separate preparations of synthesized cDNA, from 10 separate experiments to confirm the consistency of results obtained. Two different concentrations of cDNA (1 μg & 5 μg) were used for optimization of PCR amplification, so that if any isoform has a faint expression in Huh-7 cell, then could also be detected at 5 μg/μl cDNA concentration. Amplification products were separated by gel electrophoresis (2% agarose) and visualized with ethidium bromide staining. PCR products were extracted by using a QIAEX II Gel Extraction Kit (Qiagen), and sequenced by using an automated sequencer to confirm their identities.

**Table 1:** Sequences of primers used for PCR amplification of P2X receptors inHuh-7 Cells

**No**.	Primer Name	Primer Sequence: 5'-3' Sequence
1	P2X2- F	CAGGTTTGCCAAATACTACAAGATCA

2	P2X2 -R	AACTTCCCGGCCTGTCCAT

3	P2X3- F	TCTTCACCTATGAGACCACCAAGTC

4	P2X3-R	GATCAGAAGCTGAACTACTCGGTTGATG

5	P2X4 -F	CTC TGCTTGCCCAGGTACTC

6	P2X4- R	CCAGCTCACTAG CAAGACCC

7	P2X5- F	CGCTGGGGAAGCGGTTA

8	P2X5- R	GCACCAGGCAAAGATCTCACA

9	P2X6 -F	GAACCACAATTCAGCCCCTA

10	P2X6 -R	CAGGTCACAATCCCAGTGAA

11	GAPDH-F	ACCACAGTCCATGCCATCAC

12	GAPDH-R	TCCACCACCCTGTTGCTGTA

### Quantification of P2X receptors transcripts

Real time qRT-PCR was performed to examine the mRNA expression of identified isoforms of P2X receptors in Huh-7/E1E2 cell line compared to wild type/parental Huh-7 cells as control. Equal numbers of cells (3 **× **10^5^/culturing flask) of both cell lines were plated in 25 cm^2 ^culturing flasks (6culturing flasks, 1 for each cell line in 3 separate studies) at the same time and kept at 37°C incubator in humid air with 5% CO2. On 4 day, media was removed; cells were washed with sterile 1X PBS (Phosphate Buffer Saline), trypsinized using 0.5% trypsin in EDTA. The RNA extractions and cDNA symthesis were done Gentra RNA Isolation Kit (Puregene, Minneapolis, MN 55441, USA) and RevertAid™ H minus First Strand cDNA synthesis kit (Fermentas, Cat no.K1632) respectively as per manufacturer's instructions.

Quantification of P2X receptors RNAs was performed by Real Time PCR Cepheid smart cycler II (France). The relative levels of P2X2, P2X3, P2X4, P2X5, P2X6 genes were determined using GAPDH mRNA for normalization. The calculation was based on the Δ-Ct (Threshold cycle number difference between control, Huh-7, and Huh-7/E1E2 cells), and the ratios were normalized to the ratios of GAPDH of the corresponding samples. Some isoforms were unresponsive to HCV structural proteins E1E2, while some were responsive. The most responsive isoform was P2X4.

### Statistical Analysis

All statistical analysis was performed with GraphPad Prism 5 software Version 5.02. Data are presented as mean ± SEM. Comparisons between parameters were performed using ANOVA followed by Bonferroni or unpaired t test. A *p*-value of less than or equal to 0.05 was considered statistically significant.

## Results

### Identification of P2X receptors mRNA in Huh-7 cell line

**Figure 1:**
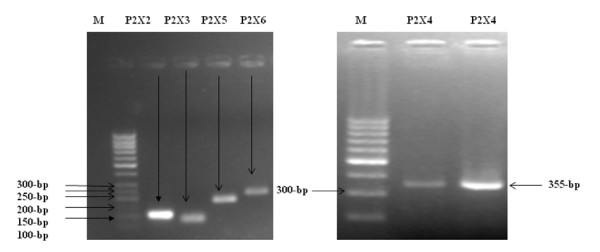
**Identification of P2X receptor transcripts in Huh-7 cell line**. Human liver cells (Huh-7 cell) express multiple P2X receptor isoform transcripts. Representative products of the appropriate size were detected for receptors P2X2, P2X3, P2X5, P2X6 and P2X4. P2X1 and P2X7 were not detected over a broad range of conditions. PCR amplifications for all detected isoforms were repeated using 10 separate preparations of synthesized cDNA, from 10 separate experiments. All these identified isoforms were always present (10/10).

To evaluate whether transcripts for P2X receptors are present in human liver cells, total cellular RNA was extracted from human hepatoma cell line (Huh-7) and was analyzed by RT PCR using primers specific for P2X1 through P2X7 (Table 1). Amplification of GAPDH mRNA served as an internal control. In whole liver, which is composed primarily of hepatocytes but also includes multiple additional cell types, mRNA for different isoforms of P2X receptors, P2X2, P2X3, P2X4, P2X5 and P2X6 were detected by RT- PCR in 10 separate studies. All studies were performed under identical culture conditions using cells from passage 5-15 and capable of forming differentiated monolayers. Figure 1 shows the detection of transcripts of different isoforms of P2X receptors in Huh-7 cell line.

Identification of amplified transcripts was confirmed by sequencing PCR. The purified DNA of P2X2, P2X3, P2X4, P2X5 and P2X6 were used as templates for sequencing PCR in the Big-Dye Terminator Cycle Sequencing Ready Reaction Kit (Applied Biosystems). Samples were analyzed on an automated sequencer (ABI PRISM 3100 genetic analyzer; Applied Biosystems). Products were sequenced from both strands to confirm their identity with reference sequences.

Sequences of the sequenced transcripts of P2X receptors were searched for homology with other sequences in GeneBank using Blast2 (Basic Local Alignment Search Tool), at http://www.ncbi.nlm.nih.gov/BLAST/ Sequences of amplified P2X receptors showed 96-100% homology with reported sequences **(**GenBank accession numbers, HQ323686, JF807485**)**.

### Expression of P2X receptors in the presence of HCV structural proteins E1E2

**Figure 2:**
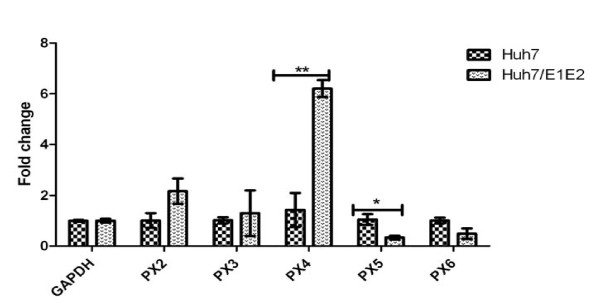
**P2X receptors genes expression analysis in Huh-7/E1E2 compared to Huh-7 cell line**. All values were expressed as mean ± SEM *P ≤ 0.05 vs. control Huh-7. Results were obtained from 6 independent experiments with replicate samples in each experiment are shown. P2X4 showed most response to HCV structural proteins E1E2.

To examine the mRNA expression of identified isoforms of P2X receptors in presence of HCV structural proteins E1E2, Real time qRT-PCR was performed. A significant increase (6.2 fold) was observed in the gene expression of P2X4 receptor in Huh-7/E1E2 cells (stably expressing HCV structural protein E1E2) in comparision with control Huh-7 cells (Figure 2). Whereas expression of P2X5 receptor decreased significantly in Huh-7/E1E2 cells (Figure 2). However, P2X2, P2X3 andP2X6 were unresponsive to E1E2.

## Discussion

Evidences showed the role of ATP as persuasive autocrine/paracrine signaling molecule in hepatocytes [21]. ATP mediates its different effects by binding to and activation of Purinergic receptors [23]. Purinergic receptors are reported to be involved in a spectrum of physiological and pathophysiological processes [24-29]. P2X receptors have previously been shown to be associated in mediating apaoptotic, inflammatory, fibrogenic and matrix deposition responses caused by various etiological factors [30].

Different isoforms of P2X receptors are expressed widely in the body [31]. Previous reports have demonstrated the presence of different transcripts of P2X receptors, P2X1, P2X2, P2X3, P2X4 and P2X7 in rat liver cells and rat hepatocytes [32]. In current study we have showed the detection of different transcripts of P2X receptors in Huh-7 and Huh-7/E1E2 cells.

The most important and interesting finding of the present study is the observation that each of these isoforms of P2X receptors responded differently to HCV structural proteins E1E2. A significant increase (6.2 fold) was observed in the expression of P2X4 receptor in Huh-7/E1E2 cells (stably expressing HCV structural protein E1E2) in comparison with control Huh-7 cells. On the other hand expression of P2X5 receptor decreased significantly in Huh-7/E1E2 cells. It has been noted in the current study that P2X2, P2X3 and P2X6 were unresponsive to E1E2. P2X4 receptors were originally identified in brain tissues where they showed unique pharmacological profile [33,34]. Previous studies provide evidence that this isoform is functionally important in hepatic functions such as regulation of hepatic glycogen metabolism in rat liver cells (Emmett, et al., 2007), in modulating biliary secretion in rat cholangiocytes [34]. There are no previous reports in literature regarding the effects of HCV proteins on the expression of P2X receptors. To the best of our knowledge the present study investigated the effects of HCV structural proteins on expression of P2X receptors for the first time.

## Conclusion

Taken together, these findings provide molecular evidence that P2X receptors are also present in human liver cells (Huh-7 cell line), and indicated that P2X receptors respond towards HCV structural proteins E1E2 of genotype 3a, P2X4 is one of the most responsive isoforms. Furthermore, these findings also suggest that the most responsive isoform P2X4 receptors could be represented a novel and functionally important component of the purinergic signaling complex in HCV induced pathogenesis. Therefore, the further additional studies also suggested for understanding the interaction of P2X receptors and HCV genome, role of P2X receptors signaling in HCV induced liver pathogenesis.

## Competing interests

The authors declare that they have no competing interests.

## Authors' contributions

SM and MI conceived the study. SM performed the all the analysis. MI and IQ helped SM in research design and performing the work. SM searched the literature and drafted the manuscript. JA, AZ, SB, KF, HA, IUR, and helped in data analysis. IQ and MI critically reviewed the manuscript. All the authors read and approved the final manuscript.
